# A particle swarm optimization improved BP neural network intelligent model for electrocardiogram classification

**DOI:** 10.1186/s12911-021-01453-6

**Published:** 2021-07-30

**Authors:** Guixiang Li, Zhongwei Tan, Weikang Xu, Fei Xu, Lei Wang, Jun Chen, Kai Wu

**Affiliations:** 1 National Engineering Research Center for Healthcare Devices, Guangdong Key Lab of Medical Electronic Instruments and Polymer Material Products, Guangdong Institute of Medical Instruments, Institute of Medicine and Health, Guangdong Academy of Sciences, Guangzhou, 510500 China; 2 Department of Biomedical Engineering, School of Material Science and Engineering, South China University of Technology, Guangzhou, 510006 China; 3 Department of Artificial Intelligence, College of Information and Communication Engineering, Hainan University, Haikou, 570228 China; 4 Guangdong Engineering Technology Research Center for Diagnosis and Rehabilitation of Dementia, Guangzhou, 510500 China; 5 Guangdong Engineering Technology Research Center for Translational Medicine of Mental Disorders, Guangzhou, 510370 China; 6 The Affiliated Brain Hospital of Guangzhou Medical University, Guangzhou Huiai Hospital, Guangzhou, 510370 China; 7 National Engineering Research Center for Tissue Restoration and Reconstruction, South China University of Technology, Guangzhou, 510006 China; 8 Key Laboratory of Biomedical Engineering of Guangdong Province, South China University of Technology, Guangzhou, 510006 China; 9 Department of Nuclear Medicine and Radiology, Institute of Development, Aging and Cancer, Tohoku University, Sendai, 980-8575 Japan

**Keywords:** Abnormal ECG identification, BP neural network, Wavelet analysis, Principal component analysis, Particle swarm optimization

## Abstract

**Background:**

As proven to reflect the work state of heart and physiological situation objectively, electrocardiogram (ECG) is widely used in the assessment of human health, especially the diagnosis of heart disease. The accuracy and reliability of abnormal ECG (AECG) decision depend to a large extent on the feature extraction. However, it is often uneasy or even impossible to obtain accurate features, as the detection process of ECG is easily disturbed by the external environment. And AECG got many species and great variation. What’s more, the ECG result obtained after a long time past, which can not reach the purpose of early warning or real-time disease diagnosis. Therefore, developing an intelligent classification model with an accurate feature extraction method to identify AECG is of quite significance. This study aimed to explore an accurate feature extraction method of ECG and establish a suitable model for identifying AECG and the diagnosis of heart disease.

**Methods:**

In this research, the wavelet combined with four operations and adaptive threshold methods were applied to filter the ECG and extract its feature waves first. Then, a BP neural network (BPNN) intelligent model and a particle swarm optimization (PSO) improved BPNN (PSO-BPNN) intelligent model based on MIT-BIH open database was established to identify ECG. To reduce the complexity of the model, the principal component analysis (PCA) was used to minimize the feature dimension.

**Results:**

Wavelet transforms combined four operations and adaptive threshold methods were capable of ECG filtering and feature extraction. PCA can significantly deduce the modeling feature dimension to minimize the complexity and save classification time. The PSO-BPNN intelligent model was suitable for identifying five types of ECG and showed better effects while comparing it with the BPNN model.

**Conclusion:**

In summary, it was further concluded that the PSO-BPNN intelligent model would be a suitable way to identify AECG and provide a tool for the diagnosis of heart disease.

## Background

Electrocardiogram (ECG) is a bio-electricity signal with low frequency and weak amplitude, objectively reflect the work state of the heart and the physiological situation, provides important information in the assessment of human health, especially for heart disease [1]. ECG is proven as the most accurate method to analyze and diagnose all kinds of arrhythmia [2]. People with cardiovascular disease usually tend to have abnormal heart rhythms in the early stages [3, 4]. If detected in real-time and find the type of abnormal heart rhythm, proceeding early warning and targeted treatment have important implications for prevention [5]. Nowadays, ECG has become a basis detection index in the clinic. In reality, ECG is one of the leading tools to assess the extent of cardiac involvement in COVID-19 patients [6]. It is of great significance to correctly identify ECG. The accuracy and reliability of ECG decision depend to a large extent on feature extraction. However, it is often uneasy or even impossible to obtained accurate features, for the ECG detection process is very easily disturbed by the external environment, and abnormal ECG (AECG) has many species and great variation. Even the same AECG of different patients also have differences. Therefore, it becomes the focus of ECG research to recognize ECG and reach the purpose of early warning or real-time disease diagnosis. Developing an intelligent classification model with an accurate feature extraction method is of quite significance to identify AECG. For ECG signal filtering, even though there is a corresponding filter bank in the system to filter out the noise in the acquisition process, the hardware denoising has certain limitations. Software de-noising mainly include three kinds of method, i.e., designing a digital filter, wavelet filter, and neural network and mathematical morphology [7–9]. In general, digital filters is difficult to design, has relatively poor execution efficiency and larger calculation. The filtering effect of the neural network is easily affected by the characteristic waveform and operation longer. For feature extraction, the detection of other characteristic waves depends on the premise of accurate detection of QRS. The method for detecting the QRS wave mainly includes mathematical morphology, difference threshold method, template matching method [10–12]. The plate matching method is based on the amplitude-frequency characteristics of the signal. The differential threshold method is simple and fast, but the detection accuracy is relatively low. The accuracy of mathematical morphology pretreatment is very high, but with complicated calculation. Overall, wavelet presents good results in signal filtering and feature extraction [13].

After feature extraction, there is remaining a big challenge for researchers to develop an intelligent and reliable system to recognize the AECG. Researchers have studied the classification method and or with a feature deduce algorithm to build a model and reduce its complication. While most ECG diagnostic systems established can not get the diagnosis result but only plays an auxiliary diagnostic role due to the complexity of ECG and difference of similar ECG. In a dynamic ECG, the classification of arrhythmia types of ECG signals has not achieved the expected real-time recognition and accurate recognition to meet clinical requirements. The analysis and recognition of ECG still need further study. A pressing problem remains in the development of an accurate method to classify AECG. In recent years, deep learning has developed rapidly, has achieved considerable progress in the research fields such as image and speech processing [14, 15]. Many scholars have begun to explore the application of deep learning methods to the detection of atrial fibrillation (AF) or other arrhythmia classification, which shows superior performance [16–24]. By using convolutional neural network (CNN), the classification accuracy only up to 83%, there remains the problem that further handle the imbalance problems of CNN frameworks and accuracy further improve [25]. The ECG classification accuracy of the model based on adversarial domain adaptation reaches 92.3% [26]. Some scholars found that the classification effect of the support vector machine method is poor, the training of linear discriminant analysis is easy to overfit, and the training time of deep learning algorithms is too long [27–29]. Scholars’ study shows that building a back-propagation neural network (BPNN) model to classify AECG, the classification accuracy is only 72.27% [30], is not suitable for detecting cardiovascular disease as the CNN. However, BPNN has strong self-learning ability and high classification and recognition ability has been used in predicting the expected cases of acquired  immune deficiency syndrome (AIDS) and shows good fitting and forecasting effects [31]. And improved BPNN also shows good effects in hand-motion recognition and water temperature forecasting [32, 33]. In this way, BPNN improved can be suitable to establish a classification model for detection AECG and cardiovascular disease, but how to design the ECG classifier based on BPNN with superior performance is worth further study.

BPNN is a multi-layer feed-forward neural network, consists of an input layer, hidden layer, and output layer. It belongs to supervised learning with the main characteristics of signal forward propagation and the error backpropagation. In general, it utilizes the difference between a theoretical value and an experimental value as a supervised signal, with an error generated in response when the output differs from that expected [34]. BPNN has the ability of simplicity, robust learning capability, and good solutions for nonlinear problems. However, it has the disadvantage of sensitivity to the weight of the initial network, is prone to a local minimum with slow convergence. And also, its lack of theoretical guidance and the selection of training samples will affect the generalization performance of the classifier [35]. Therefore, a lot of improved algorithms emerged to cope with the practical application. It mainly includes two kinds of ways, that are heuristic learning algorithm and optimization algorithm. Among them, the first method is simple and easy to use, but the performance characteristics are not very easy to set up. The other has a lot to improve the convergence speed but increased the complexity of network computing. In this study, we choose the first method. particle swarm optimization (PSO) algorithm originated from the research on artificial intelligence and the hunting behavior of birds [36]. Based on the global search strategy of the population, PSO is optimized through cooperation and competition among particles of many populations. Nowadays, PSO has been widely used in many fields, for example, function optimization, image processing, and so on [37]. Raj Sandeep and Garcia Gabriel, et al. have researched cardiac arrhythmia beat classification using PSO tuned support vector machine (SVM) and got an accuracy of 89.10% for five classes [13, 38]. Liu Zhishuai has used two-dimensionality reduction methods principal component analysis (PCA) and time window selection to get better performance in classify ECG [39]. In theory, the advantage of PSO can fill a gap of BPNN, and can be suitable to classify ECG. In this study, we mainly aimed to explore an accurate de-noising and feature extraction method of ECG based on a wavelet and perform intelligent modeling to classify AECG based on PSO optimized BPNN with combining the advantage of BPNN and PSO. With the consideration of the training time of deep learning algorithms is too long [27–29], the feature dimension reduction are also under consideration to reduce the complexity of the model to save class time and up accuracy.

## Methods

### BPNN model

**Fig. 1:**
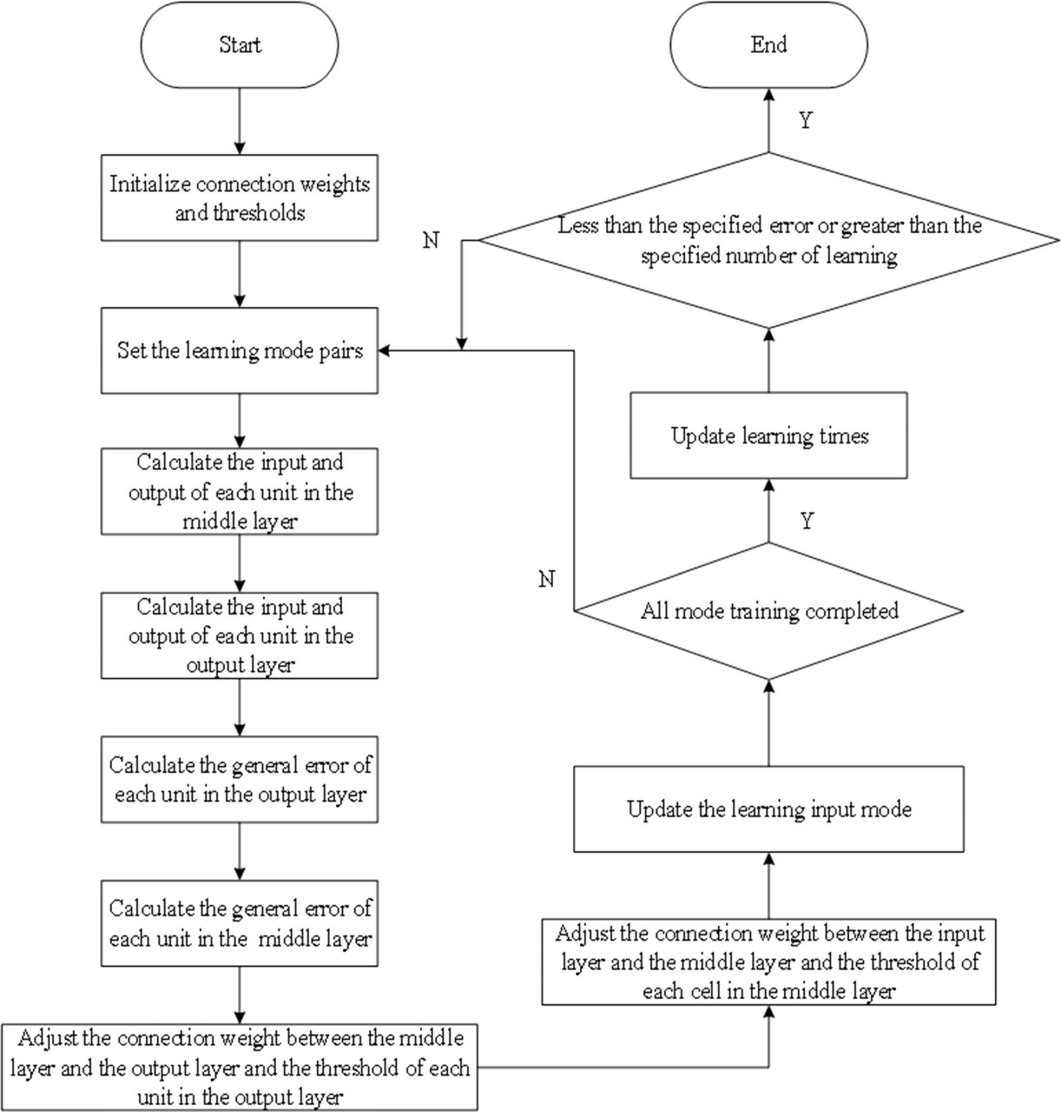
The supervised learning process of BP neural network

To a neural network (NN) model with only one hidden layer, the process of BPNN is mainly divided into two stages. The first stage is the forward propagation of the signal, which passes through the hidden layer from the input layer to the output layer. The second stage is the error backpropagation, from the output layer to the hidden layer, and finally to the input layer, in turn, adjust the weights and bias of the hidden layer to the output layer, weights, and bias of the input layer to the hidden layer. The BP learning algorithm adjusts the weight along the direction of the negative gradient, which refers to the direction in which the function goes down the fastest [40]. The learning process of BPNN is shown in Fig. 1. The weight value is revised by Eq. (1).

1 $$x_{k + 1} = x_{k} - a_{k} g_{k}$$

where $x_{k}$ is weight and threshold matrix, ${\text{g}}_{{\text{k}}}$ is the gradient of the function, $a_{k}$ is the learning rate.

The derivation analysis process is as follows [41]. Define $x_{i}$ as input layer vector, $y_{j}$ as hidden layer vector, $z_{l}$ as output layer vector, $\omega_{ji}$ as the weight vector between the input layer and hidden layer, $\nu_{lj}$ as the weight vector between the hidden layer and output layer. When the prospect output vector is *t*_1_, the output vector of the hidden layer and the output layer is given as Eqs. (2) and (3):

2 $$y_{j} = f\left( {\sum\limits_{i} {\omega_{ji} x_{i} } - \theta_{j} } \right) = f(net_{j} ),\quad net_{j} = \sum\limits_{i} {\omega_{ji} } x_{i} - \theta_{j}$$

3 $$z_{l} = f\left( {\sum\limits_{i} {\nu_{lj} y_{j} } - \theta_{l} } \right) = f(net_{l} ),\quad net_{l} = \sum\limits_{j} {\nu_{lj} } y_{j} - \theta_{l}$$

Then, the error between the expected output value and the actual output value is given as Eq. (4):

4 $${\rm E} = \frac{1}{2}\sum\limits_{l} {(t_{l} } - f(\sum\limits_{j} {\nu_{lj} } f(\sum\limits_{i} {\omega_{ji} } x_{i} - \theta_{j} ) - \theta_{l} ))^{2}$$

The derivative of the error function concerning the output vector is given as Eq. (5):

5 $$\frac{{\partial {\rm E}}}{{\partial \nu_{lj} }} = \frac{{\partial {\rm E}}}{{\partial z_{l} }} \cdot \frac{{\partial z_{l} }}{{\partial \nu_{lj} }} = - (t_{l} - z_{l} ) \cdot f^{{\prime}} (net{}_{l}) \cdot y_{i}$$

The derivative of the error function concerning the hidden vector is given as Eq. (6):

6 $$\frac{{\partial {\rm E}}}{{\partial \omega_{ji} }} = \sum\limits_{l} {\sum\limits_{j} {\frac{{\partial {\rm E}}}{{\partial z_{l} }}} } \cdot \frac{{\partial z_{l} }}{{\partial y_{j} }} \cdot \frac{{\partial y_{j} }}{{\partial \omega_{ji} }} = - \sum\limits_{l} {(t_{l} - z_{l} ) \cdot f^{{\prime}} (net{}_{l})} \cdot \nu_{lj} f^{{\prime}} (net_{j} ) \cdot x_{i}$$

The weight correction function is given as Eqs. (7) and (8):

7 $$\nu_{lj} (k + 1) = \nu_{lj} - \eta \frac{{\partial {\rm E}}}{{\partial \nu_{lj} }} = \nu_{lj} (k) + \eta (t_{l} - z_{l} ) \cdot f^{{\prime}} (net_{l} ) \cdot y_{j}$$

8 $$\begin{gathered} \omega_{ji} (k + 1) = \omega_{ji} (k) - \eta^{{\prime}} \frac{{\partial {\rm E}}}{{\partial \omega_{ji} }} \hfill \\ = \omega_{ji} (k) + \eta^{{\prime}} \sum\limits_{l} {(t_{l} - z_{l} )} \cdot f^{{\prime}} (net_{l} ) \cdot \nu_{lj} \cdot f^{{\prime}} (net_{j} ) \cdot x_{i} \hfill \\ \end{gathered}$$

$\sum\limits_{l} {(t_{l} - z_{l} ) \cdot f^{{\prime}} (net{}_{l})} \cdot \nu_{lj}$ in hidden layer node error is the error $(t_{l} - z_{l} ) \cdot f^{{\prime}} (net_{l} )$ in output node $z_{l}$ backpropagation to hidden layer node $y_{j}$ through weight $\nu_{lj}$. The corresponding threshold correction formula is given as Eqs. (9) and (10):

9 $$\theta_{l} (k + 1) = \theta_{l} (k) + \eta \frac{{\partial {\rm E}}}{{\partial \theta_{l} }} = \theta_{l} (k) + \eta (t_{1} - z_{l} ) \cdot f^{{\prime}} (net_{l} )$$

10 $$\theta_{j} (k + 1) = \theta_{j} (k) + \eta^{{\prime}} \frac{{\partial {\rm E}}}{{\partial \theta_{j} }} = \theta_{j} (k) + \eta^{{\prime}} \sum\limits_{l} {(t_{1} - z_{l} ) \cdot f^{{\prime}} (net_{l} ) \cdot \nu_{lj} } \cdot f^{{\prime}} (net_{j} )$$

where $\eta$, $\eta^{{\prime}}$ represents the learning rate of the hidden layer, the output layer, respectively.

When the transfer function is a binary type *S* function $f(x) = \frac{1}{{1 + e^{ - x} }}$, then its derivative is $f^{{\prime}} (x) = f(x) \cdot (1 - f(x))$. Therefore, $f^{{\prime}} (net_{l} )$ and $f^{{\prime}} (net_{j} )$ can be given as Eqs. (11) and (12):

11 $$z_{l} = f(net_{l} ),\quad f^{{\prime}} (net_{l} ) = z_{l} \cdot (1 - z_{l} )$$

12 $$y_{j} = f(net_{j} ),\quad f^{{\prime}} (net_{j} ) = y_{j} \cdot (1 - y_{j} ).$$

### Modeling process of BPNN

**Fig. 2:**
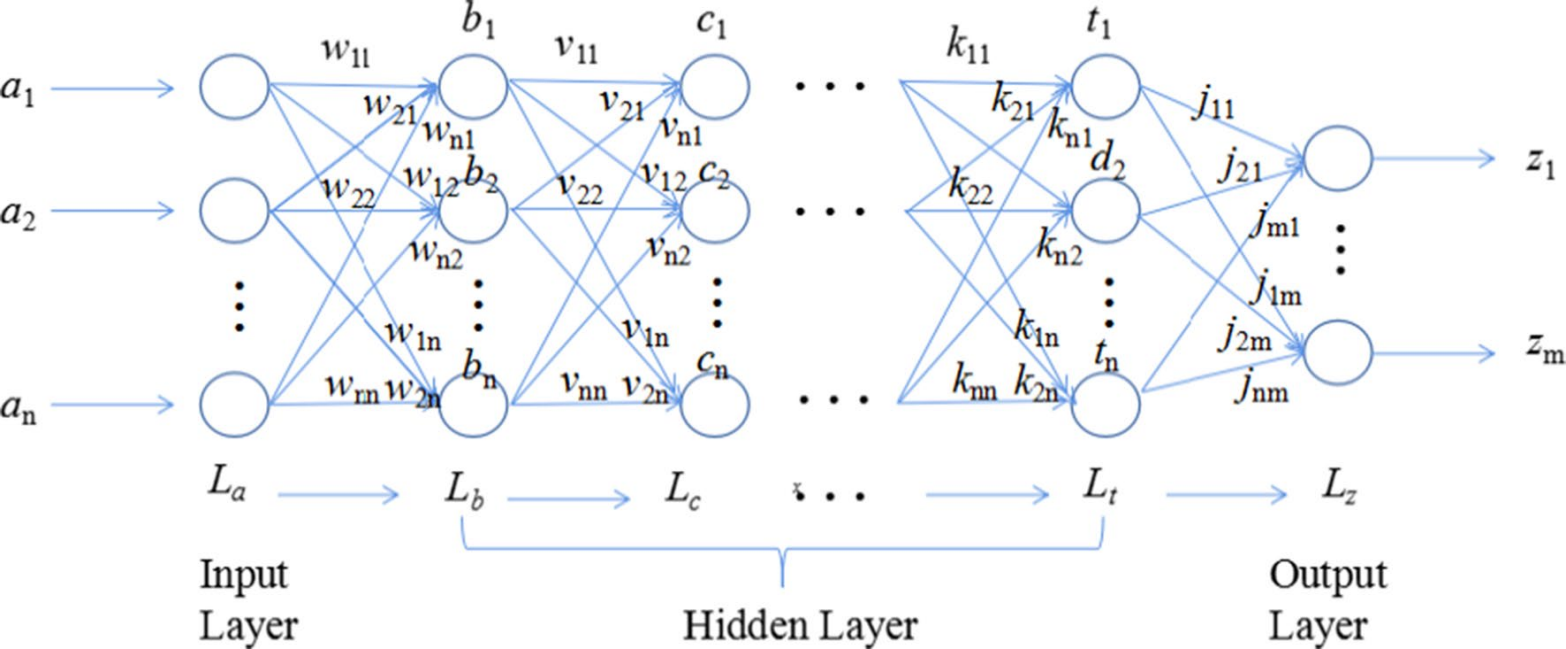
The structure of the BPNN model with multi-hidden layers

An artificial neural network (ANN) is a new cross-discipline, which is a nonlinear information processing system developed to simulate the structure and function of the human brain. A BPNN is a multilevel NN with anticipation. The most common transfer function adopted by BPNN is nonlinear transformation function (sigmoid function) in layers before the output layer, while linear function in the output layer. In a BPNN, the signal is transmitted via forwarding propagation and error by backward propagation. The BPNN is part of a multi-layered network. The most widely used ANN, the BPNN, comprises an input layer, a hidden layer, and an output layer. In this study, the structure of the BPNN model includes multi-hidden layers, as shown in Fig. 2. The process of modeling by BPNN is as follows:

1. Pre-process data.
2. Determine the number of network layers, training times, target errors, and learning speeds of the BPNN.
3. Form the BPNN with training data.
4. Use the test type data to test the NN model and finally obtain a predicted value.
5. Compare and analyze the predicted and actual values obtained in the previous step.

### Data collection and pre-process

In this study, there are five different types of MLII lead of ECG data collected to establish the classification model, from MIT-BIH open database, for MLII lead can express the changes of whole ECG well. The original ECG signal collected generally contains high-frequency noise and baseline drift. And the accuracy and reliability of AECG decision results depend to a large extent on the accuracy of ECG feature extraction.

We adopt a wavelet of common different wavelet basis functions to decompose the collected ECG signals to layer 8, to obtain the corresponding detail coefficient and approximate coefficient. From the wavelet principle, the detail coefficients of layer 1 and 2 include most of the high-frequency noise, and the approximate coefficients of layer 8 include baseline drift [42]. Therefore, we set the detail coefficient of layer 1–2 to 0 and the approximate coefficient of layer 8 to 0 to eliminate the noise. After corresponding wavelet reconstruction, we can obviously get the de-noising signal with no high-frequency noise and baseline drift. To evaluate the effects of filtering, we adopt indexes include minimum mean square error (MSE, smaller is better), signal-to-noise ratio (SNR, the closer to 1, the better), and waveform with no change to evaluate the filtering results. Based on the Matlab2019b and the first 10-s wave of record 100 ECG from the MIT-BIH arrhythmia database, the filter effects result of a wavelet of common wavelet basis function, as shown in Table 1. From the results, we found the wavelet of the sym2 basis function is the best to get smooth and pure ECG with keeping the original information, and the D-value of MSE is negligible than that of sym8.

**Table 1:** The filter effects result of a wavelet with common wavelet basis function

Basis function	Lead	Evaluate indexes	
		SNR	MSE
Haar	MLII	0.2898	4.1575e^−05^
	V5	0.2180	2.2217e^−05^
Bior2.6	MLII	1.5821	2.0868e^−05^
	V5	0.8767	1.9091e^−05^
Daubechies (db4)	MLII	0.2461	2.1572e^−05^
	V5	0.4242	4.0207e^−05^
Daubechies (db6)	MLII	1.4215	2.1942e^−05^
	V5	0.8622	1.9155e^−05^
Daubechies (db8)	MLII	0.8610	2.6452e^−05^
	V5	0.6100	2.0300e^−05^
Sym2	MLII	1.6802	3.0186e^−05^
	V5	1.1862	1.7778e^−05^
Sym4	MLII	1.2878	2.2030e^−05^
	V5	0.7427	1.9689e^−05^
Sym6	MLII	1.2881	2.2027e^−05^
	V5	0.8214	1.9225e^−05^
Sym8	MLII	1.5279	2.1191e^−05^
	V5	0.9292	1.8818e^−05^

### Heartbeat segmentation and data integration

**Fig. 3:**
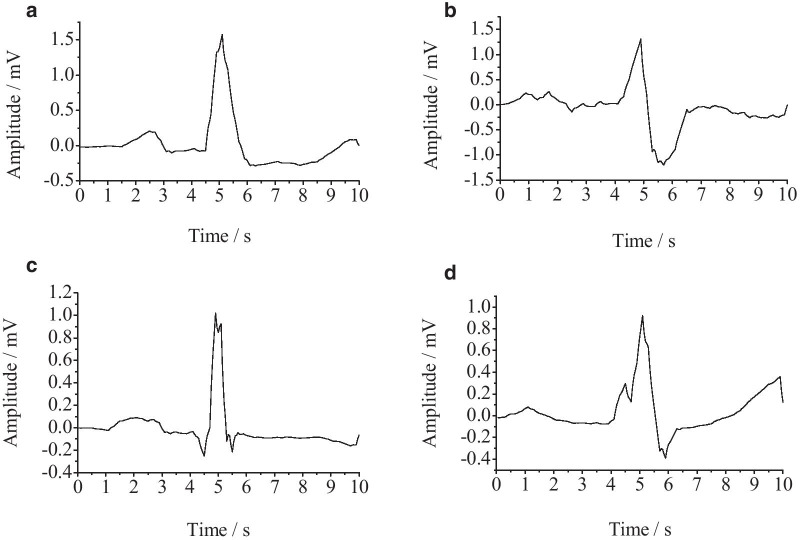
The heartbeat of four different types of AECG

As recognition of ECG mainly depends on the time difference and amplitude of the feature waves and heart rate [43, 44]. In AECG, there is always an abnormal heartbeat arise, which contains effective information for the diagnosis of heart disease. In this way, we performed cardiac beat segmentation to the collected data after filtering, according to the label. Then, combining the same labeled beats to form the type of ECG data to further treatment. The heartbeat of four different types of AECG collected was shown in Fig. 3. The Fig. 3a is the heartbeat of the ventricular premature beat (Vpb), Fig. 3b is the heartbeat of right bundle branch block (Rbbb), Fig. 3c is the heartbeat of atrial premature beats (Apb), Fig. 3d is the heartbeat of left bundle branch block (Lbbb).

Through the heartbeat segmentation, the number of five different types of ECG we collected from 23 records (records 100–233, each 30minutes) are 4615 (Lbbb), 4347 (Rbbb), 1596 (Vpb), 3016 (Apb), and 23,826 (Nb, the heartbeat of normal ECG), respectively. To minimize the calculated amount and uniform the number of beats, we selected the first 30 heartbeats into two groups to further study.

### Feature extraction

The time difference and amplitude of the characteristic waves and heart rate of ECG are the basis of diagnosis. ECG mainly includes P wave, QRS complex wave, T wave, and U wave. The normal heart rate is between 60 and 100 bpm. The P-wave represents the potential change of atrial depolarization. The PR interval represents the time when the atrium begins to depolarize, is from the beginning of the P-wave to the beginning of the QRS group. The QRS group represents the potential change of ventricular depolarization. ST-segment is the line segment from the end of the QRS group to the beginning of the T-wave, represents the process of slow ventricular repolarization. T-wave represents the potential change during rapid ventricular repolarization. QT interval represents the time required for the whole process of ventricular depolarization and repolarization, is from the beginning of the QRS wave group to the end of the T-wave. U-wave is right after the T-wave, represents the potential of ventricular follow-up. Specific changes in ECG always occur during the proceeding of disease. In clinical diagnosis and treatment, the index used to diagnosis whether the ECG is normal or not mainly includes heart rate, PR interval, QRS interval, and so on.

To obtain the diagnosis basis of ECG, accurate detection of QRS is the premise of feature extraction. To detection of QRS, the R-wave needs to be addressed first. In this study, we present a wavelet of type sym2 combined with four operations and adaptive threshold methods to perform feature extraction. We found that the energy of the R-wave is mainly contained in the detail coefficient of layer 3–5 while observing the ECG decomposed into 8 layers with wavelet sym2. Therefore, the decomposed signals at layers 3, 4, and 5 will be used for reconstruction. R-wave is oscillatory, it is still difficult to detect it after QRS composite wave is detected. Through further analysis, we found that the negative energy in the position of the Q-wave of layer 5 nearly counteracts the positive energy in the same position of layers 3 and 4. And the energy of layers 3 and 5 together nearly has the same direction of energy in layer 4. Thus, we infer that if add layers 3–5 together, the positive energy in the R-wave position can be concentrated while canceled Q-wave and S-wave that are not currently considered. And if the addition results of layers 3 and 5 multiply layer 4, there will be only positive energy left concentrated in the position of R-wave. Through experiments, we found that it is useful for making four operations to the decomposed signals at layers 3–5 to enhance R-wave by Eqs. (13) and (14):

13 $$e_{1} = d_{3} + d_{4} + d_{5} ,\quad e_{2} = d{}_{4} \times \left( {2 \times d_{3} + d_{5} } \right)$$

14 $$e_{11} = e_{1} \times e_{2}$$

where $d_{3}$ represents the decomposed signals in layer 3, $d{}_{4}$ represents the decomposed signals in layer 4, $d_{5}$ represents the decomposed signals in layer 5.

**Fig. 4:**
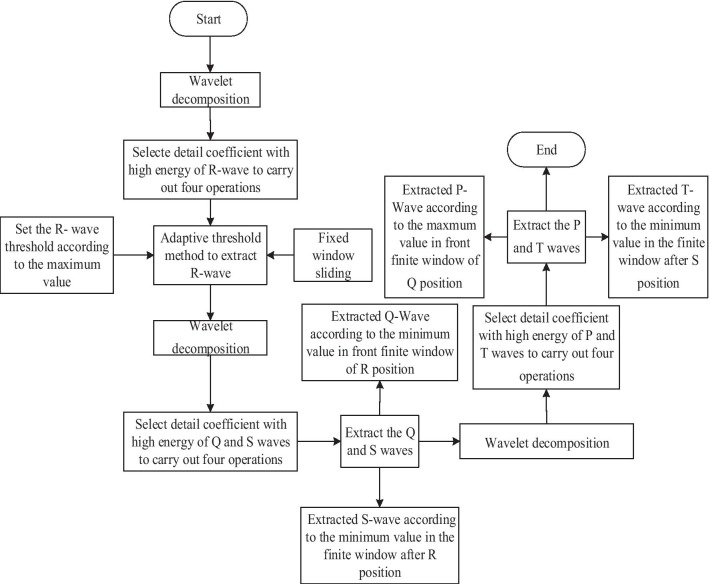
Flow chart of feature wave location

After the four operations, the energy of the R-wave enriched and other characteristic waves eliminated. In this way, only the position value of the R-wave is retained. To avoid R-wave missing and false detection, an adaptive threshold to extract R-wave was used as follows: firstly, set a fixed window with width and step length of 215 Epochs. Then, obtain the maximum value within the window and use the 60% of the maximum value as the threshold value. Lastly, sliding the window to extract the R-wave. To evaluate the extraction effect of R-wave, we use sensitivity (*Se*) and precision (*P*) to perform, by using Eq. (15). The results tested by using the first group collected data of five different types of ECG were shown in Table 2. For the *Se* and *P* of the five types of ECG are all over 93%, we think wavelet of type sym2 combined with four operations and adaptive threshold method is suitable to extract R-wave. Same as the R-wave extraction, we located all other feature waves like P-wave, Q-wave, S-wave, and T wave. Then, the time difference and amplitude of the characteristic waves and heart rate can be extracted. The flow chart of the feature wave location is shown in Fig. 4.

15 $$S{\text{e}} = \frac{TP}{{TP + FN}},\quad P = \frac{TP}{{TP + FP}}$$

where *TP* represents the number of correctly classified positive examples, i.e., it is actually a positive example and is marked positive, *FP* represents the number of falsely classified positive examples, i.e., it is actually a negative example but marked as positive, *FN* represents the number of falsely classified as negative, i.e., it is actually positive but marked as negative.

**Table 2:** The results of R-wave extracted by making adaptive discrete wavelet transform

Types	ID	*TP*	*FP*	*FN*	Sensitive (Se)	Precision (P)
Vpb	15	15	0	0	1	1
Rbbb	15	15	0	0	1	1
Nb	15	15	0	0	1	1
Apb	15	14	1	1	0.9333	0.9333
Lbbb	15	14	1	1	0.9333	0.9333

ID represents the number of heartbeats marked in the data

After all the character waves extracted, we get the feature vector consists of the time difference, amplitude, and heart rate extracted from ECG, show as Eq. (16).

16 $$F = \left[ {PQ,PR,PS,PT,QR,QS,QT,RS,RT,ST,ampP,ampQ,ampR,ampS,ampT,H} \right]$$

where *PQ* represents the time difference (TTD) between the peak values (TPV) of P-wave and Q-wave, *PR* represents TTD between TPV of P-wave and R-wave, *PS* represents TTD between TPV of P-wave and S-wave, *PT* represents TTD between TPV of P-wave and T-wave, *QR* represents TTD between TPV of Q-wave and R-wave, *QS* represents TTD between TPV of Q-wave and S-wave, *QT* represents TTD between TPV of Q-wave and T-wave, *RS* represents TTD between TPV of R-wave and S-wave, *RT* represents TTD between TPV of R-wave and T-wave, *ST* represents TTD between TPV of S-wave and T-wave, *ampP* represents TPV of P-wave, *ampQ* represents TPV of Q-wave, *ampR* represents TPV of R-wave, *ampS* represents TPV of S-wave, *ampT* represents TPV of T-wave, *H* represents the heartbeat of ECG.

Through the above procedure, the feature of 30 sets of each type of ECG was extracted. Set the feature data of each type of ECG into two groups, and then combine each group as BPNN modeling training data set and test data set. Before modeling, the data normalized. Then the BPNN model of classifying AECG was established through the process of modeling by BPNN.

### PSO-BPNN model

#### PSO algorithm

PSO algorithm originated from the research on artificial intelligence and the hunting behavior of birds [45]. Based on the global search strategy of the population, the PSO algorithm is optimized through cooperation and competition among particles of many populations [46]. Nowadays, PSO has been widely used in many fields such as function optimization, image processing, prediction, and so on, due to its simple operation, fast convergence, and global optimization capability [47, 48]. The particle swarm optimization algorithm described as follows [49]:

In an n-dimensional search space, m particles are forming a population $X = \left( {X_{1} ,X_{2} , \ldots ,X_{{\text{m}}} } \right)^{T}$, the position of the  *i-*th particle is $X_{i} = \left( {X_{i1} ,X_{i2} , \ldots ,X_{in} } \right)^{T}$, the speed is $V_{i} = \left( {V_{i1} ,V_{i2} , \ldots ,V_{in} } \right)^{T}$, the individual extreme value is $P_{i} = \left( {P_{i1} ,P_{i2} , \ldots ,P_{in} } \right)^{T}$. The global extreme value of the population is $P_{g} = \left( {P_{g1} ,P_{g2} , \ldots ,P_{gn} } \right)^{T}$. After finding the individual extreme value and the global extreme value, the particle updates its speed and position respectively according to Eqs. (17) and (18).

17 $$V_{id}^{k + 1} = V_{id}^{k} + c_{1} rand\left( {} \right)\left( {P_{id}^{k} - X_{id}^{k} } \right) + c_{2} rand\left( {} \right)\left( {P_{gd}^{k} - X_{id}^{k} } \right)$$

18 $$X_{id}^{k + 1} = X_{id}^{k} + V_{id}^{k}$$

where *c*_*1*_ and *c*_*2 *_both are non-negative constants, which are called learning factor, rand() is a random constant between (0,1), $V_{id}^{k}$ and $X_{id}^{k}$ are the velocity and position values of particle *i* in the *d* dimension in the *k*-th iteration respectively, $P_{id}^{k}$ is the position of the individual extremum of particle *i* in the *d*-dimension, $P_{gd}^{k}$ is the position of the global extreme value of the group in the *d* dimension.

#### Initial weights and thresholds optimization of BPNN

In this study, we use the PSO algorithm with an introduced speed adjustment factors to optimize the initial weight and threshold of BPNN and the conventional BP neural network model. In this way, the optimized BPNN model will have both the global optimization ability of the PSO algorithm and the local search ability of the BP algorithm.

To accelerated convergence and balance the global velocity versus local velocity of the particle, introduce inertia weight *w* and shrinkage factor *k* into the above PSO algorithm. The particle updates its speed according to Eq. (19)

19 $$\begin{aligned} V_{id}^{k + 1} & = k \cdot \left[ {wV_{id}^{k} + c_{1} rand\left( \, \right)\left( {P_{id}^{k} - X_{id}^{k} } \right) + c_{2} rand\left( \, \right)\left( {P_{gd}^{k} - X_{id}^{k} } \right)} \right] \\ k & = \frac{2}{{\left| {2 - \varphi - \sqrt {\varphi^{2} - 4\varphi } } \right|}},\quad \varphi = c_{1} + c_{2} ,\quad \varphi \ge 4 \\ \end{aligned}$$

**Fig. 5:**
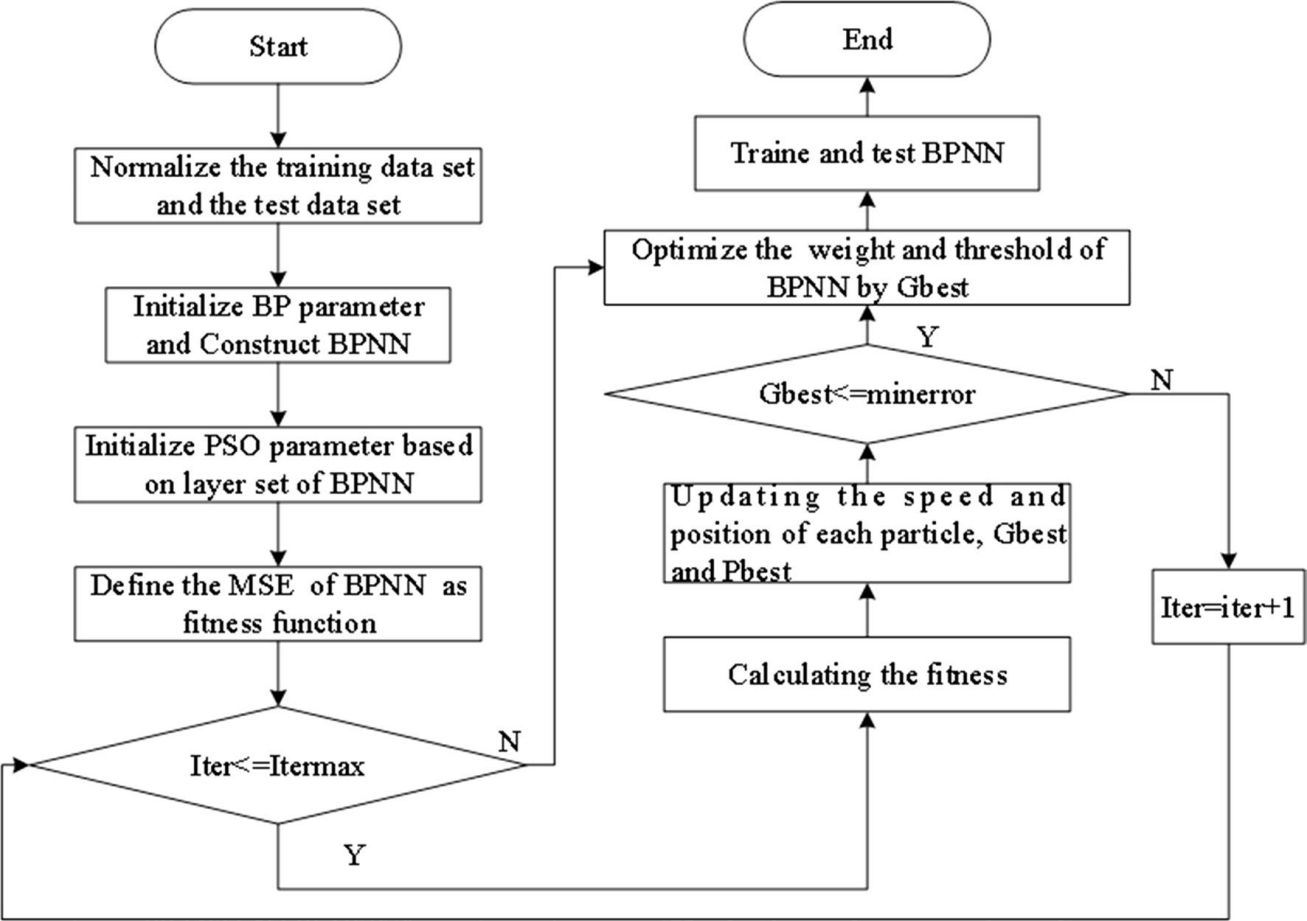
The procedure of PSO algorithm optimizing BPNN

And we use the *newff* function to create network objects:

20 $$net = newff\left( {PR,[\begin{array}{*{20}c} {S_{1} } & {S_{2} } & \cdots & {S_{N} } \\ \end{array} ],\left\{ {\begin{array}{*{20}c} {TF_{1} } & {TF_{2} } & \cdots & {TF_{N} } \\ \end{array} } \right\},BTF,BLF,PF} \right)$$

where $PR$ is $R \times 2$ matrix to define the minimum and maximum values of $R$ input vectors, $S_{i}$ express the number of layer $i$ neurons, $TF_{i}$ is the transfer function of layer $i$, the default function is tansig, $BTF$ is the training function, the default function is trainlm function, while the traingdx function is more applicable to pattern classification, $BLF$ is the weight/threshold learning function, default function is the learngdm function, $PF$ is the performance function, the default function is the MSE function. The training net and parameters are shown as follows:

- net = *newff*(pr,[5, 16],{'tansig' 'purelin'},'traingdx','learngdm'),
- pr(1:16,1) = − 1,
- pr(1:16,2) = 1,
- net.trainParam.epochs = 100,000,
- net.trainParam.goal = 0.0002,
- net.trainParam.lr = 0.0003,

The steps of the PSO algorithm optimizing the conventional BPNN is shown in Fig. 5. According to the modeling process of BPNN, we adopt the same data set for PSO-BPNN modeling. The initial and training parameters that used in modeling as shown in Table 3. In this study, the fitness function of the PSO algorithm selected the MSE of the test data set, as shown in Eq. (21):

21 $$f(x) = \frac{1}{n}\sum\nolimits_{i = 1}^{n} {\left( {{\hat{\text{y}}}_{i} - y_{i} } \right)}^{2}$$

where ¯ $\hat{y}_{i}$ is a predictive sequence and ${\text{y}}_{i}$ is a sequence of true values.

**Table 3:** The initial and training parameters

Parameters	Numerical value	Significance
indim	16	Number of input variables
hiddennum	5	Number of hidden unit
outdim	1	Number of output variables
vmax	1	Maximum velocity
minerr	0.0001	Minimum error
wmax	0.95	Maximum inertia weight
wmin	0.10	Minimum inertia weight
itermax	100	Maximum iteration number
c_1_	2.5	Local learning factor
c_2_	2.7	Global learning factor
*k*	0.37	Shrinkage factor
N	75	Number of particles
D2	116	Length of particle

## Feature dimension reduction and impact on modeling analysis

PCA is one of the most widely used data dimension reduction algorithms. Its main idea is to map the *N*-dimensional feature to the *K*-dimension, which is a new orthogonal feature, known as the principal component, and a re-constructed *K*-dimensional feature based on the original N-dimensional feature [50]. In this study, to reduce the computation and network complexity in classification, we used PCA to directly reduce the dimensions of each type of ECG heartbeat information to form the feature matrix. In theory, it is applicable to choose the dimension of accumulative contribution $a > 85\%$ [51]. To keep as much information as possible, we choose the dimension of accumulative contribution a > 85% and a > 99%. Then, use the left dimension containing nearly all the information to establish a model by using BPNN and PSO-BPNN, and to analyze the impact on modeling.

## Results

### BPNN model validation and performance analysis

**Fig. 6:**
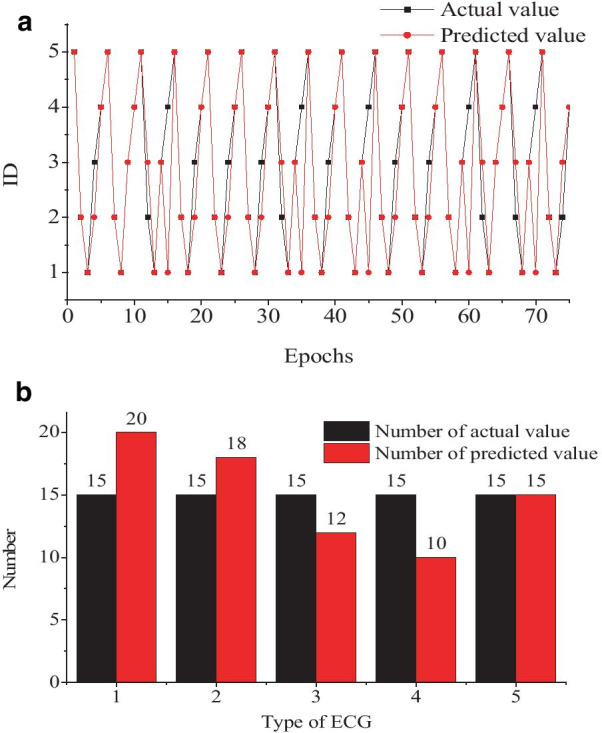
ECG classification results of the BPNN model

National Engineering Research Center for Healthcare Devices and The Guangdong Key Lab of Medical Electronic Instruments and Polymer Material Products were selected for experiments. The experiment was performed on Matlab2019b in the workstation (model: DELL 210-ANJK). To establish and evaluate a model, 30 heartbeats of feature data were collected for each of five different types of ECG collected from the MIT-BIH arrhythmia database. That is, there are 150 sets of extracted feature data. We use 75 sets of the extracted feature data to modeling, the last 75 for testing to validate the effects of the established model. When performing the experiments, we set different IDs to represent the five types of ECG, i.e., 1 represents Nb, 2 represents Lbbb, 3 represents Rbbb, 4 represents Vpb, 5 represents Apb. The results were shown in Fig. 6, in which the predicted value was round off.

To assess the accuracy of the BPNN model, we use Eq. (22) to evaluate.

22 $$Acc = \frac{{X^{{\prime}} }}{X} \times 100\%$$

where $X^{{\prime}}$ represents the number of predicted value equal to the actual value, $X$ represents the number of actual value. From the experiments, we can obtain the accuracy of the BPNN model is only 77.33%, lower than 81% of using the convolutional neural network (CNN) [52]. The time spent is 1334 s. It is not meet the practical application demand.

### PSO-BPNN model validation and performance analysis

**Fig. 7:**
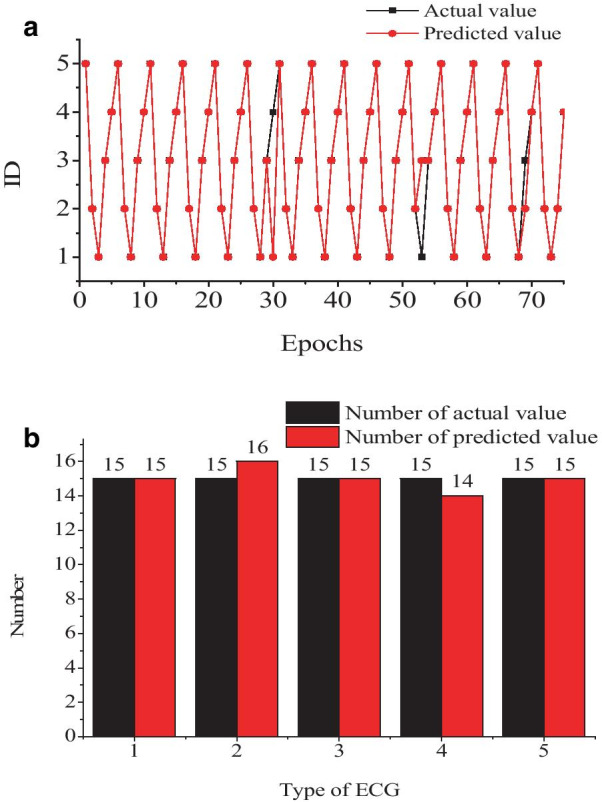
ECG classification results of PSO-BPNN model

To evaluate the PSO-BPNN model and make it a comparison to the BPNN model, we used the same data and platform. The results were shown in Fig. 7. The accuracy of the PSO-BPNN model is up to 96%. The time has been reduced to 899 s. Even the accuracy meets the practical application demand, but the time is still too long to realize a real-time diagnosis.

### Dimension reduction and impact on modeling analysis

The results of dimension reduction was shown in Table 4. We can see the when $a > 99\%$, the feature dimension reduces to half part of the original feature dimension.

**Table 4:** Dimension reduction of different accumulative contribution rates of five types of ECG

Accumulative contribution	Types	The original feature dimension	feature dimension after PCA	
			Training data	Test data
$a > 85\%$	Vpb	15	3	3
	Rbbb	15	4	3
	Nb	15	3	4
	Apb	15	4	3
	Lbbb	15	3	4
$a > 99\%$	Vpb	15	6	6
	Rbbb	15	7	6
	Nb	15	6	6
	Apb	15	7	6
	Lbbb	15	6	7

$a$ means the selected principal components can represent $a \times 100\%$ of the original information

**Fig.8:**
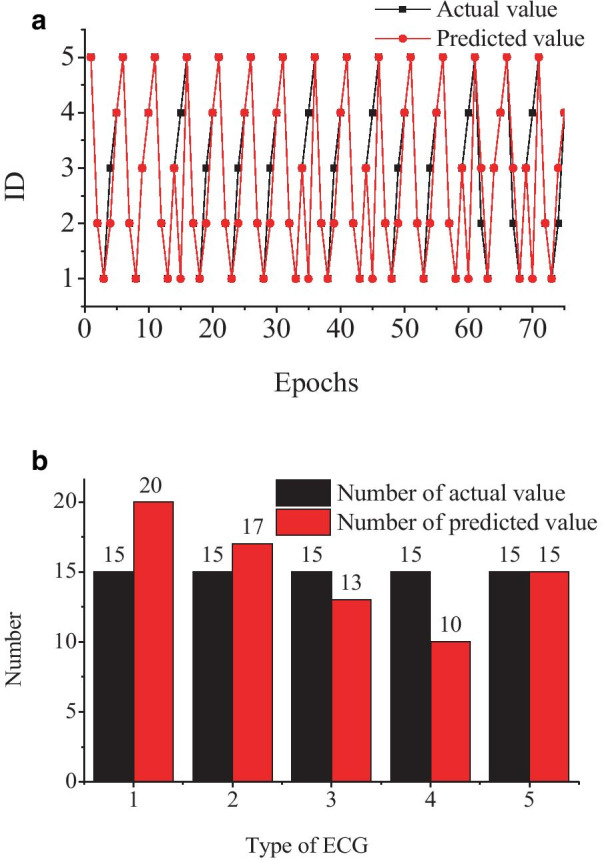
ECG classification results of the BPNN model

**Fig.9:**
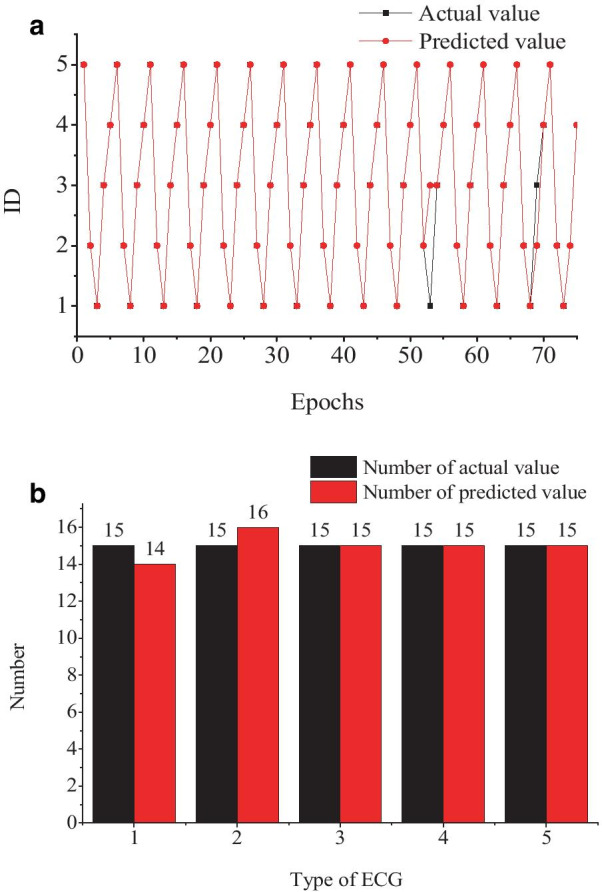
ECG classification results of PSO-BPNN model

Therefore, to analysis the impact on modeling time, seven feature dimensions were selected for modeling by BPNN and PSO-BPNN, respectively. The results are shown in Figs. 8 and 9.

The accuracy of the BPNN model is up to 80% and the time spent to cut down to 91 s. The accuracy of the PSO-BPNN model is up to 97% and the time spent to cut down to 65 s.

## Discussion

In this study, through experiments on the same platform with the same data, the PSO-BPNN model improves the accuracy and reduces the time cost, compared with the BPNN model. It indicates that the PSO algorithm has made up the defect of BPNN, which is sensitive to the weight of the initial network and prone to a local minimum. However, the classification time is still too slow to meet the actual application. The main reason could be what scholars have found, that is training time of deep learning algorithms is too long [13, 37, 38]. Except for the parameter adjustment and convergence rate of the BP neural network, the data for modeling always contained some information redundancy. In reality, too many inputs always complicate the model, which is also one reason to spend time more. To further study the reason and applicability of the PSO-BPNN model in classifying the arrhythmia. PCA, one of the feature dimension reduction algorithm was used. The reduced time and improved accuracy mean that data redundancy increases the complexity of the model. The PSO-BPNN model established in this study is suitable for ECG classification.

Several limitations still exist in this study. First of all, based on our results, future work will aim to establish data communication combined with more capable computer software to form a more complete, advanced, and simple operation. In the NN model, the study of the impact of the learning rate on the NNs has been insufficient. In general, if the learning rate is overly high, the learning process will be unstable. On the contrary, if the learning rate is too low, training will take an extended length of time. Secondly, the selection of the appropriate learning rate is the next step in optimizing the NN. The classification precision and time are analyzed qualitatively according to the given error. In future work, more test data will be added for model training to obtain a more perfect, more accurate, and more rapid soft measurement model of ECG classification.

## Conclusions

Algorithm selection has a significant impact on the accuracy of classifying results. This study employs wavelet transform combined with four operations and adaptive threshold method to perform filtering and feature extraction of ECG. Then, employ a BPNN algorithm for analysis and classification of ECG, for BPNN can make use of several optimization methods. To make up for its defect, the PSO optimization algorithm employed is extensive and expected to enhance prediction result accuracy, reduce errors incurred during experimentation owing to flawed model design, and hinder the final determination of ECG. The experiments manifest that the PSO optimized BPNN intelligent model indicating greater accuracy and better classification results than that of the conventional BPNN model.

Above all, to analyze the reason for a long time, PCA was adopted to minimize the feature dimension to re-evaluate the performance of the BPNN and PSO-BPNN model. The results show that the PCA algorithm can effectively extract the key feature dimensions and minimize the complexity of modeling. The BPNN and PSO-BPNN intelligent classification model spent less classification time but with higher accuracy. The PSO-BPNN intelligent classification model shows better effects compared with the BPNN model when identifying the five types of ECG. In conclusion, the PSO-BPNN intelligent classification model will be a suitable method to recognize ECG and provide a tool for the diagnosis of heart disease.

## Data Availability

The data used in this study are available from the MIT-BIH Arrhythmia Database (mitdb), which is one of the databases of PhysioBank ATM (https://archive.physionet.org/cgi-bin/atm/ATM). It consists of 48 half-hour ECG records from 47 subjects at Boston’s Beth Israel Hospital (now the Beth Israel Deaconess Medical Center). Each ECG data sequence has an 11 bit resolution over a 10 mV range with a sampling frequency of 360 Hz. This dataset is fully annotated with both beat-level and rhythm-level diagnoses.

## Abbreviations

ECG: Electrocardiograph
AECG: Abnormal ECG
BPNN: Backpropagation neural networks
PCA: Principal component analysis
PSO: Particle swarm optimization
NN: Neural networks
CNN: Convolutional neural network
